# Posterior reversible encephalopathy syndrome in a child with severe multisystem inflammatory syndrome due to COVID-19

**DOI:** 10.5935/0103-507X.20220028-pt

**Published:** 2022

**Authors:** Jesus Angel Dominguez-Rojas, Noe AtamariAnahui, Patrick Caqui-Vilca, Mariela TelloPezo, Pamela Muñoz-Huerta

**Affiliations:** 1 Department of Pediatric, Hospital Nacional Edgardo Rebagliati Martins - Lima, Peru.; 2 Evidencias en Salud, Universidad San Ignacio de Loyola - Lima, Peru.; 3 Department of Pediatric, Instituto Nacional de Salud del Niño - San Borja, Peru.; 4 Department of Pediatric, Instituto Nacional de Salud del Niño - Lima, Peru.

**Keywords:** COVID-19, Coronavirus infections, SARS-CoV-2, Brain diseases, Systemic inflammatory response syndrome, Child, Intensive care units, pediatric

## Abstract

Posterior reversible encephalopathy syndrome is a rare clinical and radiological syndrome characterized by vasogenic edema of the white matter of the occipital and parietal lobes, which are usually symmetrical, resulting from a secondary manifestation of acute dysfunction of the posterior cerebrovascular system. We describe a case of posterior reversible encephalopathy syndrome secondary to SARS-CoV-2 infection in a 9-year-old boy who developed acute hypoxemic respiratory failure and required assisted mechanical ventilation. The child developed multisystem inflammatory syndrome, and he was monitored in the pediatric intensive care unit and was provided mechanical ventilation and vasoactive agents for hemodynamic support. Additionally, he developed pulmonary and extrapulmonary clinical manifestations along with neuropsychiatric manifestations that required close follow-up and were verified using brain magnetic resonance imaging for timely intervention. Currently, there are few reports of children with posterior reversible encephalopathy syndrome associated with multisystem inflammatory syndrome.

## INTRODUCTION

In December 2019, a previously unknown virus emerged in the city of Wuhan, China and was associated with acute respiratory illness, ranging from mild to fulminant clinical presentations. In children, pediatric multisystem inflammatory syndrome [PIMS/multisystem inflammatory syndrome in children (MIS-C)] and other inflammatory diseases have been associated with coronavirus disease 2019 (COVID-19) during the pandemic.^(1,2)^ Little is known regarding its clinical presentation, evolution, and long-term complications. New clinical presentations associated with severe acute respiratory syndrome coronavirus 2 (SARS-CoV-2) are being discovered every day. During overactive sympathetic nervous system function, oxidative stress, endothelial dysfunction, and humoral homeostasis disorders, in particular, the interaction between a number of biochemical substances translates into clinical neurological manifestations.

## CASE REPORT

A 9-year-old boy from the city of Trujillo (Peru), previously healthy and fully vaccinated according to the vaccination schedule for his age, presented to the emergency department with respiratory distress and suspected COVID-19 infection.

The mother and child were tested for the presence of antibodies for SARS-CoV-2; the results showed that both were positive for IgG antibodies for SARS-CoV-2. In the child, the molecular reverse transcription polymerase chain reaction (RT-PCR) test for SARS-CoV-2 was negative. According to the mother, the child presented with fever, abdominal pain, diarrhea and vomiting for three days. The patient was admitted to the operating room for acute abdomen examination without further findings.

**Figure 1:**
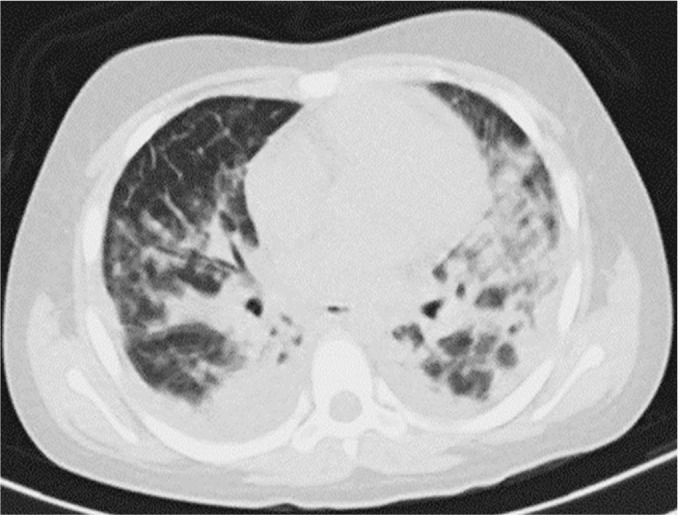
Pulmonary computed tomography performed on admission.

Twenty-four hours after surgery, the child was tachypneic and exhibited 40 breaths per minute, subcostal retractions, low oxygen saturations of 80% in room air and 85% when on high-flow oxygen support, a heart rate of 138 beats per minute and a blood pressure of 79/55mmHg. Due to his clinical deterioration, he was admitted to the pediatric intensive care unit (ICU) where he was urgently intubated for mechanical ventilation, and he presented low oxygen saturation at 85% and peripheral hypoperfusion signs. Initial venous blood gas analysis showed a pH of 7.29, partial pressure of carbon dioxide (pCO_2_) of 39.8mmHg, partial pressure of oxygen (pO_2_) of 37.3mmHg, bicarbonate levels of 17.8mmol/L, base excess of -7.8, and a 100% inspired fraction of oxygen using protective mechanic ventilation measures. Serial thoracic X-rays were performed, showing bilateral parenchymal involvement, together with a thoracic tomography scan result compatible with a consolidating parenchymal inflammatory process and bilateral pleural effusion (Figure 1).

No improvement in oxygenation was observed at the start of protective mechanical ventilation with sedoanalgesia and optimal relaxation, so it was decided to place him in the prone position for 48 hours, increasing his oxygen saturation to 92%. The child presented circulatory collapse and received several vasoactive agents (epinephrine, norepinephrine and dobutamine), achieving a mean arterial pressure within the 50th percentile for his age. The vasoactive-inotropic score (VIS) was 50, and echocardiography showed left ventricular dysfunction and vasoplegia, which reverted after 5 days with normal blood pressure figures for his age and remained so until hospital discharge.

The respiratory pathogen nucleic acid amplification panel was negative. Blood cultures, urine cultures, and peritoneal fluid samples were negative. Blood tests showed a hemoglobin level of 9.9g/dL, white blood cell count of 10,680/mm^3^, and lymphopenia (640/mm^3^). The analysis showed elevated levels of inflammatory markers, with a C-reactive protein level of 243mg/L, fibrinogen level of 410mg/dL, ferritin level of 1260ng/ mL, and hypoalbuminemia (2g/dL). In addition, the patient presented an elevated D-dimer level of 9.4mg/L with signs of coagulopathy, a prothrombin time of 49 sec, and an international normalized ratio of 1.51. Elevated prohormone B-type natriuretic peptide (282 pg/mL) and troponin (0.24pg/mL) levels were also observed, and the level of lactate dehydrogenase (LDH) was elevated. (Table 1). Fever persisted while the patient was on mechanical ventilation, and after 15 days, clinical, gasometric and radiological improvements were observed. It was possible to withdraw the patient from the mechanical ventilator to spontaneous ventilation with the support of a high-flow cannula for 30L, and the inspired oxygen fraction was 60%. After 3 weeks, the child presented neurological deterioration, with a Glasgow coma scale score of 11/15 (M4/O4/V3), pupils 3mm in diameter, presence of bilateral plantar fasciitis, delusions and suicidal ideas, psychomotor agitation, and incidence of two convulsive events of approximately 2 minutes in duration each without recovery of consciousness between events, for which he received intravenous (IV) midazolam at 0.1mg/kg and then phenytoin bolus at 20mg/kg/dose with a maintenance dose of 8mg/kg/d; this prescription was maintained upon discharge from the hospital. Psychomotor agitation was controlled with antipsychotics; 5mg of haloperidol was orally administered every 8 hours. Immunosuppressive treatment was not used.

**Table 1:** Laboratory tests performed during hospitalization

	Reference range	Onset of disease Day 0	Day 23	Day 30	Day 31
Liver function test					
Albumin (serum) (g/dL)	4 - 5.5	2 low	2.9		3.7
ALT (GPT) (serum) (U/L)	0 - 41	54		37	54
Gamma-glutamyl transferase (serum) (U/L)	11 - 50	245 high			
Glucose (mg/dL)	70 - 100	87	104	111	
Urea (mg/dL)	12 - 54	33	35	28	33
Creatinine (mg/dL)	0.7 - 1.3	0.29	0.3	0.37	0.29
Sodium (mEq/L)	< 135		137	139	
Potassium (mEq/L)	3.5 - 4		4,4	3.5	
AST (GOT) (serum) (U/L)	0 - 40	55		31	55
CK-MB Mass (serum) (ng/mL)	< 5 .0		4.1		
Troponin T (plasma) (ng/mL)	< 0.01		0.034	0.241 high	
NT-proBNP (pg/mL)	> 1				282 high
LDH (serum) (U/L	120 - 246	377 high			
Complete blood count					
HB (g/dL)	11.1 - 14.7	10.3	10.7	10	9.9 low
HCT (%)	32 - 43	30	32	30	29
Lymphocytes (/mm^3^)	1,500 - 5,000	640 low	3,540		
WBC (/mm^3^)	4,500 - 14,500	10,680	12,660	20,310	22,810 high
Platelet (/mm^3^)	200 - 550		286,000	453,000	
Serum ferritin (ng/mL)	20.0 - 300.0	1,260 high			753
PT patient (seconds)	10.8 - 12.4	18.3		12.3	
PT ratio (ratio)		13.20		13.2	
PT INR (INR)		1.51 high		0.91	
Fibrinogen (mg%)	200 - 400	410	753.5	390	
aPTT-normal (seconds)	30 - 40	31.1		30	
aPTT-patient (seconds)		36.5		31.1	
D-dimer (ng/mL)	< 0.3	3.9	9.4 high		5.1
C-reactive protei (mg/L)	< 6	243 high	87	16.3	31.4

ALT - alanine transaminase; AST - aspartate transaminase; CK-MB - creatine kinase mass; NT- pro- BNP, N- terminal pro B- type natriuretic peptide; LDH - lactate dehydrogenase; HB - hemoglobin; HCT - hematocrit ; WBC - white blood cell count; PT - prothrombin time ; INR - international normalized ratio; aPTT - activated partial thromboplastin time.

**Figure 2:**
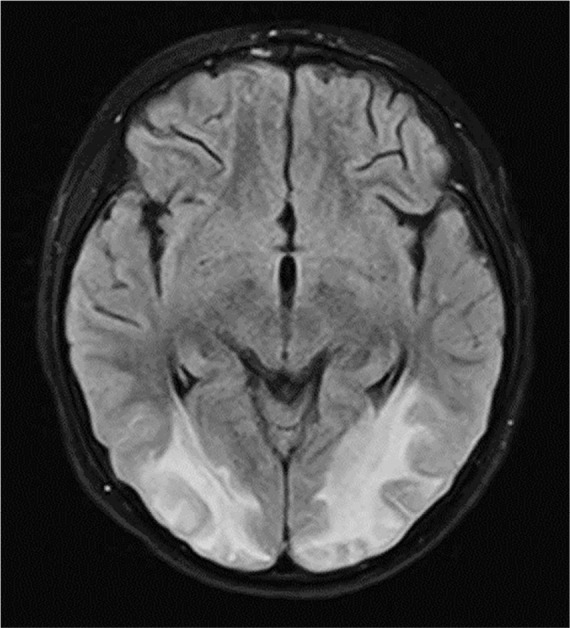
Brain magnetic resonance imaging with contrast.

Brain magnetic resonance imaging (MRI) showed bilateral occipital involvement in hyperintense T2 and FLAIR images, which symmetrically affected the subcortical white matter, especially at the posterior level, and did not stand out with contrast or show significant alterations in diffusion; these findings were compatible with vasogenic edema and indicative of posterior reversible encephalopathy syndrome (PRES) (Figure 2). Electroencephalogram findings showed generalized slowing of activity in theta/delta frequency.

To treat the multisystemic inflammatory syndrome, he received immunoglobulin at 2g/kg in 1 dose and methylprednisolone at 2mg/k/d for maintenance. Subsequently, we focused on the therapeutic management of posterior reversible encephalopathy by intravenously administering antiepileptic drugs, maintaining blood pressure levels to normal tension, maintaining adequate homeostasis of the internal environment with special emphasis on the treatment of volume overload and the avoidance of electrolyte disturbances.

During the follow-up at 4 weeks after hospital discharge, at the time of neuropsychological evaluation, skills such as attention, concentration, memory, language, visuoconstructive abilities, and calculation and orientation were assessed, and motor deficits of 4/5 in the legs were preserved. Brain MRI abnormalities were reversed 21 days after hospital discharge.

## DISCUSSION

Posterior reversible encephalopathy syndrome constitutes a transient clinical and radiological entity, with multiple risk factors in which endothelial injury and compromised cerebral perfusion are the common characteristics.

For the diagnosis of MIS-C, the available criteria were used (World Health Organization - WHO, Royal College of Pediatrics and Child Health, or Centers for Disease Control and Prevention).^(1,2)^

The patient described here with MIS-C had fever with elevated levels of inflammatory markers, such as C-reactive protein, N-terminal pro B-type natriuretic peptide, lactate dehydrogenase, D-dimer, and ferritin, and exhibited lymphopenia and low albumin levels.

Proinflammatory cytokines present in MIS-C patients, including interleukin (IL) 1β, IL-6, TNFa, and IL-17, may alter the blood-brain barrier, activate glial cells, and further instigate neuroinflammation, which causes neuronal hyperexcitability and seizures, functional alterations, fatigue, encephalopathy, loss of synapses, and even neuronal death.^(3)^ Viral interference with angiotensin 2 function in the vasculature of the central nervous system may alter the autoregulation of cerebral and systemic blood pressure.^(4)^ Moreover, vascular phenomena have also been documented in children and young adults with no medical history.^(5)^ Cerebrovascular events associated with COVID-19 in pediatric patients, including infants, should be recognized as one of the most serious presentations of COVID-19 in this patient group.^(6)^ There is limited evidence linking PRES with the presence of sepsis,^(7)^ and there is even less evidence linking it with MIS-C. The imaging features of PRES are well described, with the characteristic finding of bilateral subcortical and cortical hemispheric edema and predominance in the bilateral parieto-occipital lobes.^(8)^ Korkmazer et al. reported a case of a 10-year-old boy who developed PRES,^(9)^ and Al Haboob described a case of an 11-year-old boy,^(10)^ both with favorable evolution after established treatment. At the time of drafting this paper, we found no other published cases of COVID-19 and PRES in children. Our case is the third case reported in the literature to contribute to the knowledge and description of this entity.

In the two reported cases of acute COVID-19 and PRES in children, both had acute hypertension that would explain the PRES; however, for our phenotypic case of MIS-C and PRES, no causative agent was identified that produced alterations in cerebral autoregulation, and blood pressure was maintained in normal ranges for the patient’s age, nor were immunosuppressants used; thus, the most likely hypothesis is cerebral neuroinflammation caused by vascular hyperinflammation leading to endothelial dysfunction separate from that caused by acute COVID-19.

Our pediatric patient presented with a rare case of severe MIS-C with a varied clinical spectrum due to COVID-19 and was diagnosed with PRES based on neurological manifestations and MRI findings and treated with mechanical ventilatory support and antiepileptic therapy in the PICU. As has been shown in other reports of neurological complications in patients with COVID-19, SARS-CoV-2 may have served as a trigger for cerebrovascular endothelial dysfunction, which, in turn, was responsible for the vasogenic edema that disrupted the blood-brain barrier.

In our patient, a hyperinflammatory response to endothelial dysfunction may have been a key factor in the development of PRES.

One limitation in this clinical case was not being able to obtain cerebrospinal fluid (CSF) to perform RT-PCR molecular testing and antibodies for SARS-CoV-2 in CSF during the first days of the disease due to hemodynamic instability and the lack of specific SARS-CoV-2 testing in CSF.

## CONCLUSION

Posterior reversible encephalopathy syndrome may develop in pediatric patients with multisystem inflammatory syndrome in children and may be explained by a combination of cytokine release syndrome responsible for cerebrovascular endothelial damage.
